# Baseline Relationships between Visual Function and Inflammatory Markers in the Registry of Moderated-Stage Retinitis Pigmentosa

**DOI:** 10.1016/j.xops.2025.100930

**Published:** 2025-08-28

**Authors:** Takahiro Hisai, Sakurako Shimokawa, Masatoshi Fukushima, Kohta Fujiwara, Yoshito Koyanagi, Akie Hirata, Atsushi Takada, Fuyuka Miyahara, Naoki Nakashima, Yuko Kobayakawa, Go Mawatari, Masataka Ishizu, Naoki Toyama, Tomoko Kaida, Kazunori Miyata, Yasuhiro Ikeda, Koh-Hei Sonoda, Yusuke Murakami

**Affiliations:** 1 Department of Ophthalmology, Graduate School of Medical Sciences, Kyushu University, Fukuoka, Japan; 2 Department of Medical Informatics, Faculty of Medical Sciences, Kyushu University, Fukuoka, Japan; 3 Medical Information Center, Kyushu University Hospital, Fukuoka, Japan; 4 Center for Clinical and Translational Research, Kyushu University Hospital, Fukuoka, Japan; 5 Department of Ophthalmology, Faculty of Medicine, University of Miyazaki, Miyazaki, Japan; 6 Miyata Eye Hospital, Miyazaki, Japan

**Keywords:** Retinitis Pigmentosa, Prospective study, Registry data, Aqueous flare

## Abstract

**Purpose:**

To analyze the association between visual function and inflammatory markers in the baseline data of a prospective natural history registry of patients with typical retinitis pigmentosa (RP) (Retinitis Pigmentosa Progression and Inflammatory Marker Registry Study [RP-PRIMARY Study]).

**Design:**

A cross-sectional observational study using baseline data from the RP-PRIMARY study.

**Participants:**

A total of 67 patients with moderate-stage typical RP who were treated between October 2021 and October 2022 in 1 of 3 participating hospitals consented to participate and met the inclusion criteria.

**Methods:**

Visual functions were ETDRS best-corrected visual acuity (BCVA), Humphrey Field Analyzer 10-2 program (mean deviation value, and the mean sensitivity within the central 1° area [central 4 points, RS Cent 1’] and the 4° area [central 12 points, RS Cent 4’]), ellipsoid zone (EZ) length, central foveal thickness (CFT), hyper-autofluorescence (AF) ring area, and inflammatory markers were aqueous flare and blood test measurements.

**Main Outcome Measures:**

Association between visual function and inflammatory markers.

**Results:**

The median age of participants was 51 (interquartile range: 43–60) years. Spearman rank correlation coefficient demonstrated that aqueous flare values were negatively correlated with ETDRS BCVA (ρ = –0.35; *P* = 0.004), RS Cent 1’ (ρ = –0.32; *P* = 0.008), EZ length (ρ = –0.28; *P* = 0.023), and hyper-AF ring area (ρ = –0.31; *P* = 0.016). There was no significant correlation between systemic inflammatory markers and visual function. Eyes with intraocular lens (IOL) had significantly lower values of ETDRS BCVA (*P* = 0.004), RS Cent 1’ (*P* = 0.005), RS Cent 4’ (*P* = 0.010), CFT (*P* = 0.001), and EZ length (*P* = 0.011), in addition to higher values of aqueous flare (*P* < 0.001). Multiple linear regression analysis revealed that eyes with IOL (β = 0.262; *P* < 0.001) were significantly associated with aqueous flare.

**Conclusions:**

In the baseline data of the RP-PRIMARY study, aqueous flare, an ocular inflammatory marker, was negatively associated with visual function, and IOL implantation was most strongly associated with an increase in aqueous flare in patients with moderate-stage RP. The association between inflammatory markers and disease progression will be evaluated in the ongoing RP-PRIMARY study.

**Financial Disclosure(s):**

Proprietary or commercial disclosure may be found in the Footnotes and Disclosures at the end of this article.

Retinitis pigmentosa (RP) is a major form of inherited retinal dystrophy that causes degeneration of rod and cone photoreceptor cells.^1^ Thus far, >90 causal genes have been identified for typical RP. The clinical features of RP start with night blindness, followed by visual field constriction and central vision loss, and eventually blindness. In Japan, RP is the second most common cause of visual impairment.^2^ Neuroinflammation has been highlighted as an important biological process in RP that actively promotes or suppresses disease progression.^3^ In retrospective clinical studies, we and others previously demonstrated that ocular inflammatory markers such as aqueous flare values were negatively associated with visual function and progression of central vision loss and that expression levels of pro-inflammatory cytokines and chemokines were markedly upregulated in the aqueous humor and vitreous of RP patients.^4^, ^5^, ^6^, ^7^, ^8^ In addition, systemic inflammatory markers such as serum C-reactive protein,^9^ interleukin (IL)-8,^10^ and the CD14^++^CD16^+^ subset of inflammatory monocytes^11^ were correlated with visual function and its progression in RP patients.

Neuroinflammation in RP involves a variety of immune cell subsets, such as homeostatic microglia, disease-associated microglia, peripherally derived macrophages, and others.^3^^,^^12^^,^^13^ Although the function of each immune subset remains to be elucidated, accumulating evidence suggests that microglial phagocytosis contributes to cell death and microglial remodeling acts to protect the retina.^12^^,^^14^ In addition to these reports on inflammation and visual function in RP patients, we revealed that circulating blood inflammatory monocytes are key effector cells that mediate cone cell death in RP.^11^ Based on these results, we are currently developing anti-inflammatory pitavastatin nanoparticles (PVS-NPs) to target inflammatory monocytes to delay the progression of RP. In a preclinical study, we observed that intravenous injections of PVS-NP attenuated the activation of circulating inflammatory monocytes and protected cone function in a mouse model of RP.^11^

Elucidation of the natural history study of RP/inherited retinal dystrophies has been actively pursued to understand the prognosis and for use in clinical trials. In a pooled analysis of three randomized controlled trials, Comander et al reported that baseline electroretinogram 30 Hz flicker implicit time was a strong independent predictor of RP progression, and vitamin E had a deleterious effect.^15^ The Progression of Atrophy Secondary to Stargardt Disease study, which includes both retrospective and prospective investigations of ABCA4 mutations associated with Stargardt disease, has already released baseline data on its cohort of 365 patients with *ABCA4* gene variants and a report on the natural history of Stargardt disease over a 2-year observation period.^16^, ^17^, ^18^, ^19^, ^20^, ^21^ The Rate of Progression in usherin (USH2A)-related Retinal Degeneration study is a longitudinal investigation of patients with USH2A-associated RP and has already reported the characteristics and prognoses of these patients.^22^, ^23^, ^24^, ^25^ Collectively, these continuing analyses are providing a better understanding of the disease and assisting in the selection of patients for clinical trials. They may also provide a historical control database for such trials.

In parallel with preclinical studies of PVS-NP, we are conducting a prospective natural history study (the Retinitis Pigmentosa Progression and Inflammatory Marker Registry [RP-PRIMARY] study) for patients with RP who are eligible for future clinical trials.^26^ This is a prospective, 2-year study with follow-ups every 3 months and is designed to aid in planning future trial protocols and to serve as a historical control. In this study, we present the baseline data of the RP-PRIMARY study and analyze the association between ocular and systemic inflammatory markers and visual function.

## Methods

### Ethics Statement

This study was approved by the institutional review boards of the 3 participating hospitals (approval no. 2023–27) and was conducted in accordance with the tenets of the Declaration of Helsinki on biomedical research involving human subjects. Informed consent was obtained from all participants prior to enrollment.

### Study Design

This is a cross-sectional observational study using single time-point baseline data from the RP-PRIMARY study. The RP-PRIMARY study is a prospective and multicenter registry, with participants enrolled at three hospitals: the Kyushu University Hospital, University of Miyazaki Hospital, and Miyata Eye Hospital. The study protocol has been described in detail at https://jrct.niph.go.jp/en-latest-detail/jRCT1070210092 (jRCT1070210092) and in a protocol paper.^26^ To ensure standardized measurements and data collection, detailed procedures were outlined in the standard operation procedures and distributed to all participating sites.

The 4 visual function markers (i.e., the ETDRS best-corrected visual acuity [BCVA], intraocular pressure, and findings on spectral-domain OCT and the Humphry Field Analyzer 10-2 [HFA10-2] test, were assessed every 3 months, and the inflammatory markers aqueous flare and blood test were measured once a year. Patient characteristics data, including age, gender, disease onset, causative genes, and consanguineous marriage, were also collected at baseline, and ocular complications, ocular drug use, ocular past medical history, ocular past surgical history, height, body weight, systemic complications, dietary supplement use, smoking, and regular physical exercise were collected once a year. The inheritance pattern was determined based on the identified genetic variants.

### Patients

Patients were recruited from Kyushu University, University of Miyazaki Hospital, and Miyata Eye Hospital in October 2021 and October 2022. The diagnostic criteria for typical RP were based on the guideline of a Japanese Ministry of Health, Labour and Welfare working group^27^: progressive symptoms with a history of night blindness, photophobia, visual field constriction or ring scotoma, and markedly reduced or nonrecordable a- and b-wave amplitudes on electroretinography testing, in addition to ophthalmoscopic findings (e.g., bone spicule-like pigment clumping, macular degeneration, and attenuation of retinal vessels).

### Inclusion Criteria Were the Following

1. A diagnosis of typical RP (genetic diagnosis was unnecessary).
2. Comprehension of the design/purpose of the study and ability to declare consent.
3. Age of 20 to 70 years at the time of informed consent.
4. Mean retinal sensitivity within the central 4° area (central 12 points; RS Cent 4’) of 10 decibels (dB) or above in the HFA10-2.
5. Central foveal thickness (CFT) of ≤250 mm in the OCT measurement.

### Exclusion Criteria Were the Following

1. Anemia (Hb of ≤8 g/dl).
2. Poor general condition (performance status of ≥3).
3. Glaucoma or ocular hypertension (intraocular pressure of ≥22 mmHg.
4. Uveitis or optic neuritis.
5. Any retinal disorder not related to RP (e.g., retinal hemorrhage, retinal edema, and proliferative tissue).
6. Severe systemic complications.
7. Any other judgment of ineligibility by the investigators.

Inclusion criterion 4 was set based on our previous findings of a strong structure–function correlation in the 4° area (RS Cent 4’) in RP patients and a nonlinear structure–function relationship in RP patients with RS Cent 4’ <10 dB.^28^ Inclusion criterion 5 was based on another study by our group in which RP patients with CFT >252 μm showed faster progression than patients with CFT <252 μm.^29^

In a prior retrospective study, RS Cent 4’ was shown to decline at a rate of –0.78 dB/year in RP patients with CFT of ≤252 μm.^29^ Based on this finding, we performed a sample size calculation assuming that a new treatment reduces the progression rate by 30%. This yielded a sample size estimate of 170 patients for a two-arm study (n = 85 per group). Considering an anticipated 10% dropout rate, we set our target sample size at 100 patients in the RP-PRIMARY study.

The examination results of 1 eye that met all five eligibility criteria for each subject were used in the analyses. In cases where both eyes met the inclusion criteria, priority was given to the eye without a history of cataract surgery and without macular complications such as epiretinal membrane or cystoid macular edema. If both eyes were comparable in these respects, the eye with the better mean deviation (MD) on the HFA10-2 was selected.

### Visual Acuity

Visual acuity was measured with the ETDRS number chart (no. 2702A, Precision Vision, Inc), placed at a 4 m distance, after refractive correction.

### Visual Field Testing

Static automated perimetry using the HFA10-2 Swedish Interactive Thresholding Algorithm Standard program was conducted before mydriasis. The lens was corrected as appropriate for the test distance. This program delivers a stimulus dynamic range of 0 to 50 dB, with a background luminance of 31.5 asb and a maximum luminance of 10 000 asb. The test uses the standard Goldmann III stimulus size and a duration of 200 ms. Visual field testing was repeated if the test reliability was not satisfactory (fixation loss >20%, false positive >15%, or false negative >33%). Mean deviation values were provided in the examination report by averaging the differences between the measured sensitivities and the age-adjusted normal sensitivities (total deviations) at each test point. RS Cent 1’ (central 4 points) and RS Cent 4’ (central 12 points) were calculated as described previously.^6^^,^^29^, ^30^, ^31^

### CFT, EZ Length, and Hyper-AF Ring Area

OCT images were acquired using a spectral-domain OCT device (Spectralis, Heidelberg Engineering). Horizontal and vertical retinal scans were recorded, and >50 scans were averaged for each subject.

For measurement of CFT, the center thickness value in the retinal map analyzed by Heidelberg Retina Angiograph-OCT software was used. However, if there was a difference of ≥10 μm between the center and central minimum values, CFT was measured using the caliper tool on horizontal or vertical scans. The measurement line was drawn perpendicular to the tangent line of the foveal retinal pigment epithelium (RPE), and the length from the foveal inner limiting membrane to the base of the RPE was calculated. For measuring the ellipsoid zone (EZ) length, the distal points of the EZ were defined as the locations where the EZ was disrupted and met the RPE line. Using the straight caliper tool of the Heidelberg Retina Angiograph-OCT software, a measurement line was placed along the tangent of the foveal RPE, and the length between the EZ disruption points was then calculated. If the normal EZ was not present, EZ length was determined to be 0 mm.

A hyper-autofluorescence (AF) image was also obtained with the spectral-domain OCT device (Spectralis) with a 55° objective lens. The hyper-AF ring area was measured in patients with a normal AF density in the central fovea and a surrounding hyper-AF ring. Using the caliper tool in the Heidelberg Retina Angiograph-OCT software, the outer edge of the AF ring was traced. If there was no central normal AF area, the hyper-AF ring area was recorded as 0 mm^2^. If the normal AF area exceeded the measurement area, the distal end of the image was traced instead.

### Laser Flare Photometry

The aqueous flare was measured with a Kowa FM-600 laser flare meter (Kowa Co). After administering 0.5% tropicamide and 0.5% phenylephrine hydrochloride for mydriasis, 5 measurements were taken and averaged in each eye.

### Blood Testing

All samples were subjected to a blood cell count (hemogram) and biochemistry test in each hospital. For IL-8 and monocyte subset analysis, the blood samples (serum and isolated monocytes, respectively) were aliquoted within 1 hour of blood draw and stored at –80°C and then transferred to the laboratory of the Department of Ophthalmology, Kyushu University.

Measurement of IL-8 was performed with the multiplex enzyme-linked immunosorbent assay (ELISA)-based Q-Plex Human Cytokine array (Quansys Biosciences). The array was used according to the manufacturer's instructions and analyzed in duplicate. The average value of 2 tests was adopted for each patient, and if the coefficient of variation was >10, the measurement was repeated.

Flow cytometry analysis of monocyte subsets was performed on a CytoFLEX S (Beckman Coulter). Six antibodies were used for classification: CD2, CD14, CD16, CD19, CD56, and HLA–DR isotype. FACSVerse and FlowJo software (version 10.9.0; BD Biosciences) were used to analyze the classical CD14^++^CD16^-^, intermediate CD14^++^CD16^+^, and nonclassical CD14^+^CD16^++^ monocytes.

### Statistical Analysis

The data are presented as the arithmetic median (interquartile range). The relationship between inflammatory markers and visual parameters was examined by Spearman rank correlation coefficient. Given that some of the measurement values did not follow a normal distribution, the data were analyzed by Mann–Whitney *U* test for comparison of values and analyzed by Fisher exact test for comparison of rates. For examining the association of aqueous flare with characteristics, the data were analyzed by a multivariate linear regression analysis using clinically relevant variables: age, gender, intraocular lens (IOL) implantation, ETDRS BCVA, and EZ length. Aqueous flare values were transformed into logarithms to approach normal distribution in linear regression analysis. All of the statistical analyses were performed with RStudio software (version 2024.12.1+563; Posit). *P* values <0.05 were considered significant.

## Results

### Baseline Characteristics

Among 80 patients who agreed to participate in the RP-PRIMARY study, 67 patients met the inclusion criteria. The demographic data of the study patients are summarized in Table 1 (Table S1, available at www.ophthalmologyscience.org, shows the data for each hospital). The median age of the patients was 51 (interquartile range: 43–60) years, and 37.3% were male. The median onset age was 27 (interquartile range: 15–40) years old. Causative genes were identified in 46.3% of patients. Among these, the inheritance pattern was autosomal dominant in 4.5% (3/67 patients), autosomal recessive in 40.3% (27/67), and X-linked recessive in 1.5% (1/67). The causative genes were not identified in 40.3% of patients (27/67), and 13.4% (9/67) did not undergo genetic testing. The most frequent causative gene was *EYS* (25.4%: 17/67 patients), followed by *USH2A* (7.5%: 5/67 patients). Table 1 and Table S1 also show ocular characteristics of the patients. Fourteen patients had previous cataract surgery, and three patients had previous vitrectomy combined with cataract surgery. All of these surgeries were conducted at least 1 year before enrollment. In 3 patients, cystoid macular edema was associated with RP, and two of them received dorzolamide eye drop treatment. The designation “others” under eye drops included dry eye and eye strain. No patients received either steroid eye drop or systemic steroid therapy.

**Table 1:** Patient Characteristics in the RP-PRIMARY Study

Variable	Value
Patient	67
Age, yrs	51 (43, 60)
Male, n	25 (37.3%)
Right eye, n	22 (32.8%)
Disease onset, yrs	27 (15, 40)
Inheritance mode and causative gene, n	
AD	3 (4.5%)
*RHO*	1
*SNRNP200*	1
*TOPORS*	1
AR	27 (40.3%)
*EYS*	17
*USH2A*	5
*PDE6B*	2
*ABCA4*	1
*PDE6A*	1
*PROM1*	1
XR	1 (1.5%)
*RPGR*	1
Not determined	27 (40.3%)
Not tested	9 (13.4%)
Consanguineous marriage, n	9 (13.4%)
BMI, kg/m^2^	22.5 (20.8–25.1)
Systemic past history, n	
Diabetes mellitus	2 (3.0%)
Hyperlipidemia	10 (14.9%)
Hypertension	15 (22.4%)
Ischemic heart disease	2 (3.0%)
Dietary supplement, n	
Vitamin A	4 (6.0%)
DHA	5 (7.5%)
Lutein	5 (7.5%)
Combination	3 (4.5%)
Ex- and current smoker, n	18 (26.9%)
Regular exercise habit, n	31 (46.3%)
Lens, n	
Cataract	28 (41.8%)
Intraocular lens	14 (20.9%)
Macular complication, n	
ERM	6 (9.0%)
CME	3 (4.5%)
Intraocular surgery, n	
Cataract surgery	14 (20.9%)
Vitrectomy	3 (4.5%)
Eyedrops, n	
Dorzolamide	8 (12.0%)
Isopropyl	6 (9.0%)
Bromfenac	1 (1.5%)
Others	20 (29.9%)

AD = autosomal dominant; AR = autosomal recessive; BMI = body mass index; CME = cystoid macular edema; DHA = docosahexaenoic acid; ERM = epiretinal membrane; RP-PRIMARY = Retinitis Pigmentosa Progression and Inflammatory Marker Registry Study; USH2A = usherin; XR = X-linked recessive.

Values are given as median (interquartile range) or number.

Table 2 shows the median values of visual parameters and inflammatory markers. ETDRS BCVA was 74.0 (interquartile range: 62.5–79.5) letters, and the median MD value was –16.2 (interquartile range: –21.4 to –13.2) dB. The median EZ length (horizontal) was 0.89 (interquartile range: 0.36–1.81) mm, and the hyper-AF ring area was 0.0 (interquartile range: 0.0–3.1) mm^2^; there were 15 patients with a 0 mm EZ line and 46 patients with a 0 mm^2^ hyper-AF ring area. One patient did not undergo hyper-AF ring area measurement because of an equipment malfunction. The aqueous flare value was 9.0 (interquartile range: 6.9–14.6) pc/ms. Median serum IL-8 was 12.0 (interquartile range: 9.2–15.4) pg/ml, and the median classical, intermediate, and nonclassical monocyte ratios were 83.8% (interquartile range: 78.3–86.9%), 7.4% (interquartile range: 5.5–10.0%), and 8.0% (interquartile range: 6.1–11.5%), respectively. One patient did not undergo flow cytometry analysis of monocyte subsets because of an equipment malfunction.

**Table 2:** Median Values of Inflammatory Markers and Visual Parameters

Variable	Value
ETDRS BCVA, letters	74.0 (62.5–79.5)
IOP, mmHg	13.0 (11.0–15.0)
HFA10-2, dB	
MD	–16.2 (–21.4 to –13.2)
RS Cent 1’	28.3 (21.9–31.6)
RS Cent 4’	24.4 (19.6–27.6)
CFT, μm	186.0 (154.0–216.5)
EZ length, mm	
Horizontal	0.89 (0.36–1.81)
Vertical	1.06 (0.45–1.71)
Hyper-AF ring area, mm^2^∗	0.0 (0.0–3.1)
Aqueous flare, pc/ms	9.0 (6.9–14.6)
Hs-CRP, mg/dl	0.05 (0.02–0.09)
Serum IL-8, pg/ml	12.0 (9.2–15.4)
CD14/CD16 subsets, %∗	
CD14^++^CD16^-^ classical	83.8 (78.3–86.9)
CD14^++^CD16^+^ intermediate	7.4 (5.5–10.0)
CD14^+^CD16^++^ nonclassical	8.0 (6.1–11.5)

AF = autofluorescence; BCVA = best-corrected visual acuity; CFT = central foveal thickness; dB = decibels; EZ = ellipsoid zone; HFA10-2 = Humphrey Field Analyzer 10-2; hs-CRP = high-sensitivity C-reactive protein; IL-8 = interleukin-8; IOP = intraocular pressure; MD = mean deviation; RS Cent 1'/4' = mean retinal sensitivity within the central 4/12 points in HFA10-2.

Values are given as median (IQR) (n = 67).

∗ n = 66.

### Correlation between Inflammatory Markers and Visual Function

**Figure 1:**
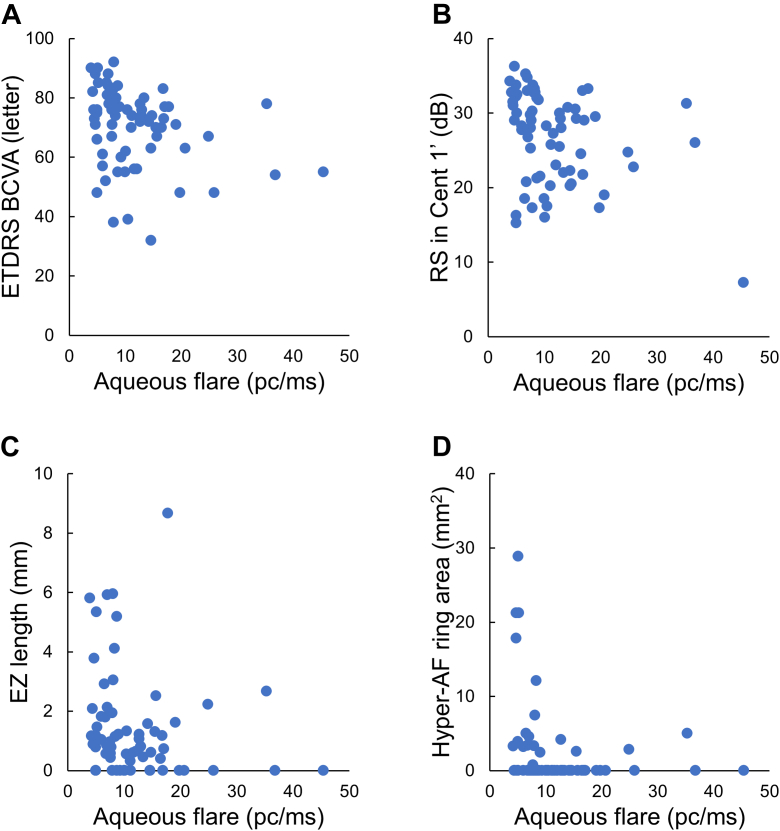
Correlations between aqueous flare values and visual parameters. (**A**–**D**) Scatter plot of aqueous flare values and 4 different visual parameters: ETDRS BCVA (**A**), RS Cent 1’ (**B**), horizontal EZ length (**C**), and hyper-AF ring area (**D**). The Spearman rank correlation coefficient showed a significant correlation between aqueous flare and ETDRS BCVA (ρ = –0.35; *P* = 0.004), RS Cent 1’ (ρ = –0.32; *P* = 0.008), EZ length (ρ = –0.28; *P* = 0.023), and hyper-AF ring area (ρ = –0.31; *P* = 0.016). BCVA = best-corrected visual acuity; EZ = ellipsoid zone; AF = autofluorescence.

The correlation between aqueous flare values and the indices of visual function was analyzed by Spearman rank correlation coefficient. The aqueous flare value was negatively correlated with ETDRS BCVA (ρ = –0.35; *P* = 0.004), RS Cent 1’ (ρ = –0.32; *P* = 0.008), EZ length (horizontal: ρ = –0.28; *P* = 0.023; and vertical: ρ = –0.35; *P* = 0.004), and hyper-AF ring area (ρ = –0.31; *P* = 0.016) (Fig 1 and Table 3). Although the systemic inflammation markers—serum high-sensitivity C-reactive protein (hs-CRP), IL-8, and monocyte subsets—showed weak correlations with some visual and structural parameters, none of these associations reached statistical significance (all *P* > 0.100). The detailed correlation coefficients and *P* values are provided in Table S2 available at www.ophthalmologyscience.org.

**Table 3:** Correlation Coefficient Analyses between Aqueous Flare Value and Visual Parameters

Variable	Aqueous Flare	
	ρ	*P* Value
ETDRS BCVA	–0.35	**0.004**
MD	–0.14	0.245
RS Cent 1’	–0.32	**0.008**
RS Cent 4’	–0.22	0.070
CFT	–0.15	0.213
EZ length (horizontal)	–0.28	**0.023**
EZ length (vertical)	–0.35	**0.004**
Hyper-AF ring area	–0.31	**0.016**

AF = autofluorescence; BCVA = best-corrected visual acuity; CFT = central foveal thickness; EZ = ellipsoid zone; MD = mean deviation; RS Cent 1'/4' = mean retinal sensitivity within the central 4/12 points in HFA10-2.

Bold values indicate statistically significant results (*P* < 0.050).

### Effects of Lens Status on Ocular Inflammation and Visual Status

**Figure 2:**
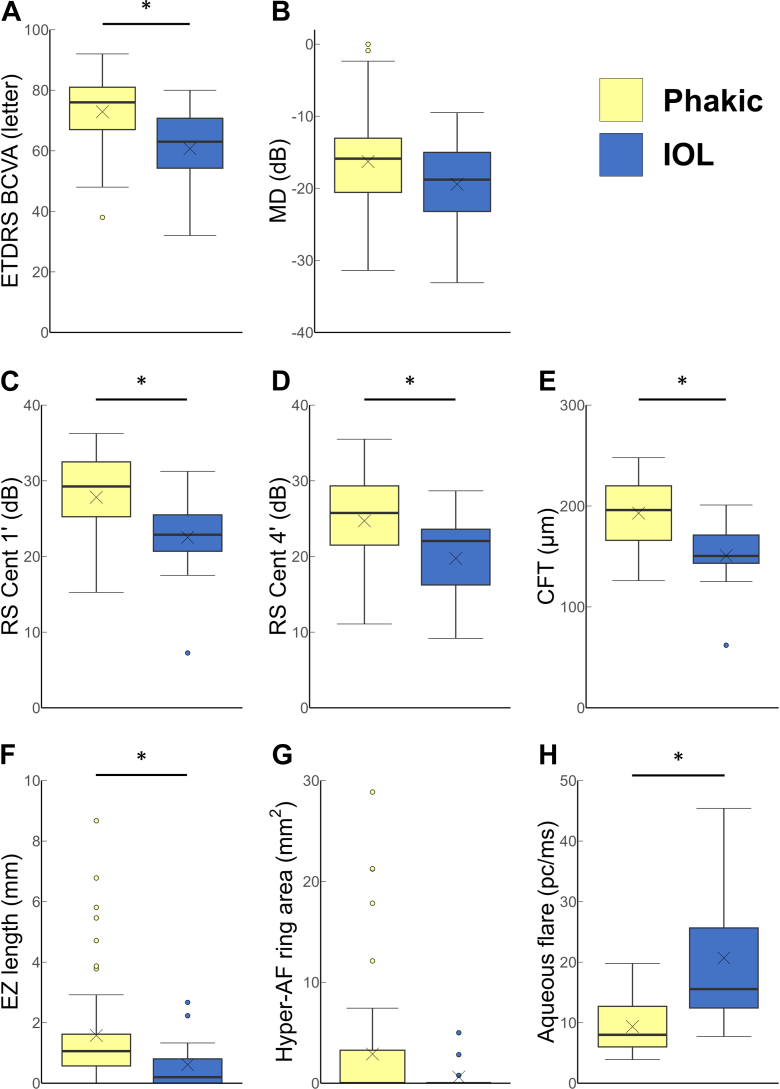
Comparison of visual functional parameters between phakic and IOL eyes. (**A**–**H**) Differences in ETDRS BCVA (**A**), MD (**B**), RS Cent 1’ (**C**), RS Cent 4’ (**D**), CFT (**E**), EZ length (**F**), hyper-AF ring area (**G**), and aqueous flare values (**H**) between phakic (yellow bars) and IOL (blue bars) eyes. The × mark represents the mean, and the box plot indicates, from bottom to top, the upper quartile (Q1), median (Q2), and lower quartile (Q3). The whiskers represent the minimum and maximum values or the data range within 1.5 × IQR (IQR = Q3 – Q1). Outliers beyond the whiskers' range are shown as individual points. Statistical differences were analyzed by the Mann–Whitney *U* test. ∗Statistically significant at *P* < 0.05. AF = autofluorescence; BCVA = best-corrected visual acuity; CFT = central foveal thickness; EZ = ellipsoid zone; IOL = intraocular lens; IQR = interquartile range; MD = mean deviation.

Since cataract surgery can affect visual function, we next compared aqueous flare values and indices of visual function between phakic eyes and eyes with IOL (Table 4 and Fig 2). Phakic eyes had significantly higher values of multiple visual function parameters: ETDRS BCVA (phakic: 76.0 [interquartile range: 67.0–81.0]; IOL: 63.0 [interquartile range: 54.3–70.8]; *P* = 0.004), RS Cent 1’ (phakic: 29.3 [interquartile range: 25.3–32.5] dB; IOL: 22.9 [interquartile range: 20.7–25.5] dB; *P* = 0.005), RS Cent 4’ (phakic: 25.8 [interquartile range: 21.5–29.3] dB; IOL: 22.0 [interquartile range: 16.2–23.6] dB; *P* = 0.010), CFT (phakic: 196.0 [interquartile range: 166.0–220.0] μm; IOL: 150.5 [interquartile range: 143.3–171.3] μm; *P* = 0.001), horizontal EZ length (phakic: 1.06 [interquartile range: 0.58–1.94] mm; IOL: 0.20 [interquartile range: 0.00–0.75] mm; *P* = 0.011), and vertical EZ length (phakic: 1.25 [interquartile range: 0.65–1.92] mm; IOL: 0.21 [interquartile range: 0.00–0.79] mm; *P* = 0.003). In addition, aqueous flare values were approximately twice as high in IOL eyes (15.6 [interquartile range: 12.4–25.7] pc/ms) compared with phakic eyes (8.0 [interquartile range: 6.0–12.7] pc/ms) (*P* < 0.001). Patients with IOL eyes were older than those with phakic eyes (phakic: 50.0 [interquartile range: 42.0–56.0] years; IOL: 61.0 [interquartile range: 51.8–67.0] years; *P* = 0.013). To account for the potential influence of age on visual function, we performed multiple regression analyses adjusting for age. After adjustment, phakic eyes still showed significantly better visual function across multiple parameters (all *P* < 0.05), except for horizontal EZ length (*P* = 0.071). Additionally, aqueous flare values remained significantly higher in IOL eyes (*P* < 0.001).

**Table 4:** Comparison of Visual Parameters between Phakic and IOL Eyes

Variable	Phakic (n = 53)	IOL (n = 14)	*P* Value (Crude)	*P* Value (Age-Adjusted)
Age, yrs	50.0 (42.0–56.0)	61.0 (51.8–67.0)	**0.013**	-
ETDRS BCVA, letter	76.0 (67.0–81.0)	63.0 (54.3–70.8)	**0.004**	**0.005**
MD, dB	–15.9 (–20.6 to –13.1)	–18.8 (–23.2 to –15.0)	0.335	-
RS Cent 1’, dB	29.3 (25.3–32.5)	22.9 (20.7–25.5)	**0.005**	**0.009**
RS Cent 4’, dB	25.8 (21.5–29.3)	22.0 (16.2–23.6)	**0.010**	**0.026**
CFT, μm	196.0 (166.0–220.0)	150.5 (143.3–171.3)	**0.001**	**<0.001**
EZ length (horizontal), mm	1.06 (0.58–1.94)	0.20 (0.00–0.75)	**0.011**	0.071
EZ length (vertical), mm	1.25 (0.65–1.92)	0.21 (0.00–0.79)	**0.003**	**0.041**
Hyper-AF ring area, mm^2^	0.0 (0.0–3.3)∗	0.0 (0.0–0.0)	0.232	-
Aqueous flare, pc/ms	8.0 (6.0–12.7)	15.6 (12.4–25.7)	**<0.001**	**<0.001**

AF = autofluorescence; BCVA = best-corrected visual acuity; CFT = central foveal thickness; dB = decibels; EZ = ellipsoid zone; IOL = intraocular lens; MD = mean deviation; RS Cent 1'/4' = mean retinal sensitivity within the central 4/12 points in HFA10-2.

Values are given as median (interquartile range).

Bold values indicate statistically significant results (*P* < 0.050).

∗ n = 52.

### Ocular and Systemic Factors Associated with Aqueous Flare Values

We next analyzed ocular and systemic factors associated with aqueous flare (Table 5). In the univariate analysis, age (β = 0.008; *P* = 0.002), IOL implantation (β = 0.330; *P* < 0.001), ETDRS BCVA (β = –0.006; *P* = 0.008), and vertical EZ length (β = –0.040; *P* = 0.024) were significantly associated with log-transformed aqueous flare values. To further evaluate whether these were independently associated with log-transformed aqueous flare values, we conducted a multivariate linear regression analysis. The model included age, IOL implantation, and gender, along with ETDRS BCVA or EZ length to account for disease severity. In Adjusted-1, ETDRS BCVA was included, whereas in Adjusted-2, vertical EZ length was used. In both models, IOL implantation remained significantly associated with aqueous flare values (β = 0.262; *P* < 0.001 [Adjusted-1]; β = 0.264; *P* < 0.001 [Adjusted-2]), whereas the association with age and disease severity was no longer significant. These results suggest that elevated aqueous flare in IOL eyes was independent of age and disease severity.

**Table 5:** Characteristics and Aqueous Flare Values in Multiple Linear Regression

Variable	Aqueous Flare Values (per 1-Log–Transformed)					
	Crude		Multiple Linear Regression			
			Adjusted-1		Adjusted-2	
	β (95% CI)	*P* Value	β (95% CI)	*P* Value	β (95% CI)	*P* Value
Age	0.008 (0.003–0.012)	**0.002**	0.004 (–0.000 to 0.009)	0.057	0.004 (–0.000 to 0.009)	0.058
Gender	0.119 (–0.001–0.238)	0.052	0.036 (–0.068 to 0.141)	0.489	0.042 (–0.062 to 0.146)	0.425
IOL	0.330 (0.209–0.452)	**<0.001**	0.262 (0.128–0.396)	**<0.001**	0.264 (0.133–0.395)	**<0.001**
ETDRS BCVA	–0.006 (–0.010 to –0.002)	**0.008**	–0.002 (–0.006 to 0.002)	0.370	-	-
EZ length (horizontal)	–0.027 (–0.060 to 0.006)	0.107	-	-	-	-
EZ length (vertical)	–0.040 (–0.074 to –0.006)	**0.024**	-	-	–0.016 (–0.046 to 0.014)	0.296

BCVA = best-corrected visual acuity; EZ = ellipsoid zone; IOL = intraocular lens.

Adjusted-1: Age, gender, IOL, and ETDRS adjusted; Adjusted-2: Age, gender, IOL, and EZ length (vertical) adjusted.

Bold values indicate statistically significant results (*P* < 0.050).

## Discussion

The baseline data of the RP-PRIMARY study showed that aqueous flare, an ocular inflammation marker, were correlated with the visual parameters ETDRS BCVA, RS Cent 1’, EZ length, and hyper-AF ring area. On the other hand, systemic inflammatory markers (hs-CRP, IL-8, and monocyte subsets) were not associated with baseline visual function.

Our previous retrospective studies demonstrated that aqueous flare values were significantly correlated with BCVA and with the slope of MD, RS Cent 1’, and RS Cent 4’ in patients with RP.^5^^,^^6^ Nishiguchi et al also reported a correlation between aqueous flare and visual field area using Goldmann perimetry.^8^ Specifically, they confirmed a significant correlation between aqueous flare and functional visual parameters, with a correlation coefficient of –0.35 between aqueous flare and ETDRS BCVA (*P* = 0.004), which was comparable to our previous findings (aqueous flare and logarithm of the minimum angle of resolution BCVA; ρ = 0.359; *P* < 0.0001). In addition to functional visual parameters, our present study indicated that EZ length and hyper-AF ring area were correlated with aqueous flare. Because EZ and fundus autofluorescence are established indicators of central visual function in RP patients,^32^, ^33^, ^34^, ^35^, ^36^ these correlations suggest that aqueous flare may serve as a biomarker of RP severity. However, because this study employed a cross-sectional design, it cannot establish causality between inflammation and visual function. It remains unclear whether increased intraocular inflammation (e.g., aqueous flare) contributes to a decline in visual function, or vice versa. This limitation will be addressed in the ongoing RP-PRIMARY, which will track temporal changes and explore potential causal relationships.

In contrast, no significant correlation was found between systemic inflammation markers (e.g., hs-CRP, IL-8, and monocyte subsets) and visual function. This contradicts our previous findings linking systemic inflammation and visual function in RP.^10^ One possible explanation is that our patient population was older than the <40-year-old^9^^,^^10^ or <45-year-old^11^ populations in prior studies. Systemic factors related to aging and lifestyle diseases may have confounded the results. Another possibility is that systemic inflammatory markers are more closely associated with disease progression rather than severity, as hs-CRP^9^ and the ratio of the CD14^++^CD16^+^ subset of inflammatory monocytes^11^ correlated with the decline rate of MD but not with the baseline MD values. Therefore, the relationships between systemic inflammation and disease progression of RP should be evaluated in the ongoing prospective studies.

Patients who underwent cataract surgery had significantly worse visual function and higher aqueous flare values than those without surgery. Previously, we reported that visual acuity improved 6 months after cataract surgery in RP patients, particularly in those with a preserved foveal EZ.^37^ However, in many cases, visual function declined to a level similar to or worse than preoperative values approximately 6 years after surgery.^38^ In contrast, De Rojas et al found no association between cataract surgery and EZ-loss progression.^39^ Although the causal relationship remains unclear, aqueous flare values were approximately twice as high in eyes with IOL, and IOL implantation was identified as a significant predictor of elevated aqueous flare in the multivariate linear regression analysis. Because elevated aqueous flare levels can persist for up to 3 years postcataract surgery,^40^ chronic inflammation may contribute to visual decline. Alternatively, as high aqueous flare values are linked to a higher frequency of posterior subcapsular cataract,^41^ these patients may have undergone earlier cataract surgery. Taken together, these findings suggest a relationship between cataract surgery, intraocular inflammation, and vision loss. Causal relationships cannot be determined from a cross-sectional analysis; further clarification will require longitudinal evaluation in the ongoing prospective study.

Because our investigational drug (PVS-NP) targets the common pathological feature of neuroinflammation in RP, the RP-PRIMARY study enrolled patients with typical rod-cone dystrophy, regardless of their genotypes. The genetic diagnosis rate was 46.3%, and 10 different causative genes were identified, with EYS being the most frequent (25.4%; 17/67 patients). These results align with those of Goto et al, who investigated the genotypes of 2325 Japanese RP patients (solved rate: 38.6%; *EYS* frequency: 18.0%),^42^^,^^43^ suggesting that our cohort may be representative of the general Japanese RP population. Previous reports indicated better visual functions in autosomal dominant cases compared with autosomal recessive cases.^1^^,^^44^^,^^45^ However, in the present study, because of the limited number of genetically confirmed autosomal dominant cases (n = 3), we did not perform statistical comparisons across inheritance patterns.

This study has several limitations. First, although we initially planned a sample size of 100 patients, patient recruitment was significantly affected by the coronavirus disease 2019 pandemic, and it was not feasible to include additional sites, resulting in a final sample size of 67 patients. Secondly, blood test results may be affected not only by the RP pathology but also by the systemic conditions and complications. The baseline data were collected during the coronavirus disease 2019 epidemic period, and it may be possible that patients’ immunological status was affected by viral infection or vaccine treatment. Thirdly, the study population may not fully represent the broader RP patient population. The inclusion and exclusion criteria—particularly the exclusion of patients with CFT >250 μm and RS Cent 4′ <10 dB—resulted in a cohort skewed toward individuals at a moderate disease stage and a relatively faster progression rate. This selection bias limits the generalizability of the findings to the wider RP population. Despite these limitations, this registry has the advantage of providing high-quality data comparable to that in clinical trials because it includes standard operation procedures, case report forms, and monitoring by an independent certified investigator. The system was reviewed by the Pharmaceuticals and Medical Devices Agency, the relevant Japanese regulatory agency, to ensure the quality of the data and its potential use in future clinical trials.

In conclusion, aqueous flare, an ocular inflammatory marker, was negatively associated with visual parameters in the baseline data of the RP-PRIMARY study. In contrast, systemic inflammatory markers did not correlate with the baseline visual function. The associations between inflammatory markers and disease progression will be evaluated in the ongoing prospective study, and these data will be utilized to develop anti-inflammatory drugs aimed at slowing disease progression and preventing blindness in RP.
