# Supplementary material for: Baseline Relationships between Visual Function and Inflammatory Markers in the Registry of Moderated-Stage Retinitis Pigmentosa
Source: Ophthalmol Sci. 2025 Aug 28;6(1):100930. doi: 10.1016/j.xops.2025.100930 (PMC12553051; doi:10.1016/j.xops.2025.100930)
Supplement: Table S1 [file mmc1.pdf]

Table S1. Patient Characteristics at Participating Facilities in the RP-PRIMARY Study

	KUH	UMH	MEH	**All**
**Patient**	46	12	9	**67**
**Age, y.o.**	53 (43, 60)	46 (42, 52)	51 (48, 67)	**51 (43. 60)**
**Male, n**	17 (37.0%)	4 (33.3%)	4 (44.4%)	**25 (37.3%)**
**Right eye, n**	13 (28.2%)	7 (58.3%)	2 (22.2%)	**22 (32.8%)**
**Disease onset, y.o.**	25 (14, 40)	25 (16, 42)	34 (30, 40)	**27 (15, 40)**
**Inheritance mode and causative gene, n**				
AD	3 (6.5%)			**3 (4.5%)**
RHO	1			**1**
SNRNP200	1			**1**
TOPORS	1			**1**
AR	22 (47.8%)	2 (16.7%)	3 (33.3%)	**27 (40.3%)**
EYS	16		1	**17**
USH2A	2	1	2	**5**
PDE6B	2			**2**
ABCA4		1		**1**
PDE6A	1			**1**
PROM1	1			**1**
XR		1 (8.3%)		**1 (1.5%)**
RPGR		1		**1**
Not Determined	17 (37.0%)	6 (50.0%)	4 (44.4%)	**27 (40.3%)**

Not Tested	4 (8.7%)	3 (25.0%)	2 (22.2%)	**9 (13.4%)**
**Consanguineous marriage, n**	7 (15.2%)	0 (0%)	2 (22.2%)	**9 (13.4%)**
**BMI, kg/m^2**	23.0 (21.1. 25.0)	20.9 (19.9, 23.8)	21.9 (21.0, 29.2)	**22.5 (20.8, 25.1)**
**Systemic past history, n**				
Diabetes mellitus	1 (2.2%)		1 (11.1%)	**2 (3.0%)**
Hyperlipemia	7 (15.2%)	2 (16.7%)	1 (11.1%)	**10 (14.9%)**
Hypertension	8 (17.4%)	4 (33.3%)	3 (33.3%)	**15 (22.4%)**
Ischemic heart disease	1 (2.2%)		1 (11.1%)	**2 (3.0%)**
**Dietary supplement, n**				
Vitamin A	3 (6.5%)	1 (8.3%)		**4 (6.0%)**
DHA	4 (8.7%)		1 (11.1%)	**5 (7.5%)**
Lutein	4 (8.7%)		1 (11.1%)	**5 (7.5%)**
Combination	3 (6.5%)			**3 (4.5%)**
**Ex- and current smoker, n**	13 (28.3%)	2 (16.7%)	3 (33.3%)	**18 (26.9%)**
**Regular exercise habit, n**	21 (45.7%)	5 (41.7%)	5 (55.6%)	**31 (46.3%)**
**Lens, n**				
Cataract	25 (54.3%)	2 (16.7%)	1 (11.1%)	**28 (41.8%)**
Intraocular lens	8 (17.4%)	1 (8.3%)	5 (55.6%)	**14 (20.9%)**
**Macular complication, n**				
ERM	4 (8.7%)	2 (16.7%)		**6 (9.0%)**
CME	1 (2.2%)	2 (16.7%)		**3 (4.5%)**
**Intraocular surgery, n**				
Cataract surgery	8 (17.4%)	1 (8.3%)	5 (55.6%)	**14 (20.9%)**
Vitrectomy	2 (4.3%)	1 (8.3%)		**3 (4.5%)**
**Eyedrops, n**				

Dorzolamide	6 (13.0%)	1 (8.3%)	1 (11.1%)	**8 (12.0%)**
Isopropyl	2 (4.3%)		4 (44.4%)	**6 (9.0%)**
Bromfenac			1 (11.1%)	**1 (1.5%)**
Others	17 (37.0%)	1 (8.3%)	2 (22.2%)	**20 (29.9%)**

Values are given as median (IQR) or number.

KUH: Kyushu University Hospital; UMH: University of Miyazaki Hospital; MEH: Miyata Eye Hospital; AD: autosomal dominant; AR: autosomal recessive; XR: X-linked recessive; BMI: body mass index; DHA: docosahexaenoic acid; ERM: epiretinal membrane; CME: cystoid macular edema.
