# Supplementary material for: Baseline Relationships between Visual Function and Inflammatory Markers in the Registry of Moderated-Stage Retinitis Pigmentosa
Source: Ophthalmol Sci. 2025 Aug 28;6(1):100930. doi: 10.1016/j.xops.2025.100930 (PMC12553051; doi:10.1016/j.xops.2025.100930)
Supplement: Table S2 [file mmc2.pdf]

Table S2: Correlation Coefficient Analyses Between Systemic Inflammatory Markers and Visual Parameters

	hs-CRP		IL-8		$CD14^{++}CD16^{+}$	
	ρ	P value	ρ	P value	ρ	P value
ETDRS BCVA	-0.05	.704	0.10	.439	-0.01	.906
MD	-0.17	.160	0.05	.710	-0.15	.223
RS Cent 1’	-0.04	.742	0.01	.943	-0.11	.366
RS Cent 4’	-0.06	.637	0.09	.493	-0.12	.336
CFT	-0.16	.204	0.04	.745	-0.09	.496
EZ length (horizontal)	-0.05	.675	-0.17	.163	-0.18	.153
EZ length (vertical)	-0.04	.767	-0.13	.299	-0.17	.172
Hyper-AF ring area	-0.05	.680	-0.15	.237	-0.21	.101

hs-CRP: high-sensitive C-reactive protein; IL-8: interleukin-8; ETDRS BCVA: early treatment diabetic retinopathy study best-corrected visual acuity; MD: mean deviation; RS Cent 1'/4': mean retinal sensitivity within the central 4/12 points in HFA10-2; CFT: central fovea thickness; EZ: ellipsoid zone; AF: autofluorescence.
